# Assessing the performance of Hungary’s primary health care system during the COVID-19 pandemic: findings from the cross-sectional PRICOV-19 study

**DOI:** 10.1186/s12875-026-03429-9

**Published:** 2026-07-08

**Authors:** Zoltán Lakó-Futó, Krisztián Vörös, Esther van Poel, Sara Willems, Péter Torzsa

**Affiliations:** 1 Department of Family Medicine, Faculty of Medicine, Semmelweis University, Budapest, Hungary; 2 Department of Public Health and Primary Care, Ghent University, Ghent, 9000 Belgium

**Keywords:** Covid19, Primary care, Physicians' wellbeing, Adoptation to pandemic, Teaching practice, Telemedicine

## Abstract

**Background:**

Primary care (PC) has played a crucial role in the fight against the COVID-19 epidemic. The novel challenges have significantly increased the workload for PC workers, leading to negative impacts on their mental health. Hungarian primary care is in the early stage of moving from solo practices to primary health care teams, while the number of elderly physicians increases quickly. The PRICOV-19 study is a multicenter study in 38 countries that comprehensively assesses the performance of PC during the pandemic. This article focuses on the impact of COVID-19 epidemic on resilience and adaptability of PC in Hungary, during the time of structural change.

**Methods:**

The questionnaire was sent online to PC practices between April and August 2021 as email links. The 53 questions of the survey were structured around six themes: infection control, patient flow, information acquisition (protocols and knowledge acquisition), patient communication, collegial cooperation, and quality of life of the care workers. To measure the well-being of care workers, the validated Mayo Clinic Wellbeing Index was used.

**Results:**

Respondents of the study reported notable limitations to their work environment (91, 4%). In response to these challenges, there was a significant increase in implementation of telemedicine rising from 15.3% before COVID to 36.7%. Almost all PC workers reported a substantial surge in their workload during the pandemic (96.7%). Furthermore, many of them (37.8%) expressed the need for additional training to manage the unprecedented challenges posed by the pandemic. These new tasks had a worrisome impact on PC workers, with 55.2% of respondents expressing concern about the increased workload, and 69% experienced some degree of distress. Longer work experience was associated with better adaptation and better quality of life.

**Conclusion:**

The COVID-19 pandemic brought a substantial change in primary car in Hungary, in terms of patient flow, information exchange and communication. While the feeling of being not prepared and lower well-being was common among GPs, adaptation in task shifting and protocol driven telemedicine appeared, especially among more experienced physicians. The process of adjustment in challenging times could be fostered by unambiguous central measurements and through adequate training.

## Background

The outbreak of the COVID-19 epidemic posed a new challenge to healthcare systems worldwide [1, 2]. In Hungary, the first restrictions were announced on March 15, 2020, which affected all areas of social contact, the whole spectrum of healthcare, including primary care (PC). The cloud-based medical services (drug prescriptions) that had just been introduced at the time, and had been operated parallel with the traditional paper-based systems, practically became dominant during the closures [3–5]. Face-to-face consultations gave way to telemedicine care, primarily telephone consultations, but other online structures (email, messaging services) also became very popular [6, 7]. Epidemic prevention had a clear priority; plannable health care services had been postponed [8, 9]. Accordingly, the majority of inpatient departments were closed, giving space to departments providing care for COVID-19 patients. The older medical colleagues primarily took on the role of telemedicine care, while the younger colleagues were transferred to the wards caring for those infected with COVID-19.

PC took the lion's share in this anti-epidemic care-optimized system [9–11]. Following the frequently, sometimes weekly changing epidemiological directives, general practitioners (GPs) and GP nurses treated and cared for patients convalescing at home, while trying to comply with the also expected curative and preventive PC activities [12, 13]. In the early period, especially at the time of the first wave/closure (spring 2020), there was a lack of adequate amount of protective equipment and disinfectant [14–16], moreover inappropriate amount and quality of COVID-19 tests available for the health sector. The lockdown during the first wave was very strict and managed to keep the number of active cases low to give time for healthcare to prepare. The newly introduced cloud-based service [17] was an effective help when starting telemedicine services [18], but due to the short preparation period, many providers used this application uncertainly.

Later, in early 2021, with the arrival of vaccines against COVID-19, a new front was opened in the fight against the epidemic [19]. The administration of vaccines and the organization of candidates' transportation to vaccination points became the task of PC. The situation was further complicated by the presence of vaccines of different origins and backgrounds. In Hungary, in addition to vaccines purchased by the European Union, Chinese (Sinopharm) and Russian (Sputnik V) vaccines were also available [20]. Various directives were issued regarding the indications of different vaccines [21], considering the age and co-morbidities of the applicants. In addition, the preferences of the candidates also had to be considered [22]. In this sensitive situation, GPs had to organize and partly carry out the administration of vaccinations.

The increased physical and psychological stress during the epidemic had a significant impact on the mental health of healthcare workers [23, 24] in general, and also PC workers [25, 26], characterized by an age well beyond society's mean age [27], with a high proportion of elderly doctors and nurses [28]. Generalized anxiety, depression, sleep and attention disorders, combined with reduced work capabilities and fatigue, and a feeling of joylessness, were the typical symptoms of the healthcare workers. All of these effects were studied primarily on inpatient care workers [2, 15, 16, 25, 29–34], while we have little data on the mental health of PC colleagues during the COVID-19 pandemic.

The Pricov-19 study investigated the status of workers in PC during the COVID-19 pandemic in 38 countries, including Hungary. The study examined the perceptions of PC providers in patient care, infection prevention and control, patient follow-up and communication, and collaboration, with particular attention to the rapid, forced introduction of telemedicine care, the role of educational practices, and the preservation of mental health.

Hungarian primary care is in the early stage of moving from solo practices to primary health care teams. The mean age of GPs is above 60 years, caring for an aging population. Thus, organizational challenges might differ from that of other countries. While focusing on the well-being of current GPs is important, creating a structure that is attractive for young doctors is also necessary. In this study we aimed to describe the changes COVID imposed on Hungarian primary care, and to identify workflow challenges and factors that may endanger the well-being of general practitioners.

## Methods

The Hungarian results were obtained as part of the PRICOV-19, a cross-sectional multicenter study covering 38 countries. Changes in PC during the pandemic and the adaptation of PC workers to the challenges posed by the coronavirus epidemic were the focal points of the PRICOV-19 study. A detailed description of the methodology can be found here [35]. In short: the questionnaires developed by Ghent University and sent in English were translated into Hungarian and forwarded to family medicine colleagues working in PC through the REDCap software [36].

Two translators (KV and ZLF) independently provided the initial translation of the questionnaire. This version was synthesised, then reverse translated back to English (PT). After comparing the back-translation to the original questionnaire, the translators agreed by consensus on the final version. The few sentence structure and semantic differences in translations were resolved during the synthesis. Three coworker physicians at the Department of Family Medicine tested the questionnaire to make sure it was comprehensible and without misunderstandings. No cultural adaptation was necessary in Hungary.

Similarly to half of the countries participating in the PRICOV study, data gathering was carried out according to the National Convenient Sampling method. We sent emails to GPs who had previously consented to receiving messages from us, including teaching GPs or physicians who had participated in our continuous medical education programs. We reached out to 315 colleagues through our mailing list and asked them to fill out the questionnaire and to forward the link to the questionnaire to 5 of their previous fellow physicians (chain sampling). Of the 315 GPs 127 sent out further 635 emails, thus altogether 950 emails were delivered. We received answers from all areas of the country and from all types of settlements the practices were located in, nevertheless the sampling was not representative. Out of the 950 colleagues, 222 responded (23.4%) and 165 (17.4%) filled out the questionnaire completely.

The questionnaires were answered in Hungary between April and August 2021, on the REDCap site, managed by the researchers of Ghent University.

The questionnaire contained 53 questions, divided into six themes for both practice and employee data: infection control, patient flow data before and during the epidemic, information acquisition (protocols and knowledge acquisition), patient communication, collegial cooperation, employee quality of life. Employee well-being was assessed based on the validated Mayo Clinic Wellbeing Index [37] (MCWI).

The MCWI measures dimensions of distress, namely burnout and emotional detachment, depression, fatigue, anxiety/stress, and physical exertion, and asks two questions assessing meaning in work and satisfaction with work-life integration. The first 7 items are yes/no questions scored as 0 or 1. The last two questions are answered on a Likert scale of 1 to 7. Answers for these questions are converted to binary scores: 1 and 2 answers (strongly and very strongly disagree) are scored to 1 point, the neutral answers of 3 to 5 scored to 0, while the 6 and 7 answers (strongly or very strongly agree) are scored to –1. Sum scores range from the maximal well-being of –2 (all No answers on the first 7 questions and positive opinion on work meaning and work-life integration) to 9 points (all Yes answers on the first 7 questions and negative opinion on work meaning and work-life integration).

The staff of Ghent University carried out data cleaning. The consortium partners carried out further processing of the national data.

Data is presented as percentage and number for categorical, and mean ± standard deviation for continuous variables. Correlation of ordinal variables was assessed by rank correlation analysis (Spearman). The effect of predictors on well-being (MCWI) was evaluated using backward multivariate logistic regression analysis. The Mayo index was categorized at the cut-off point of less than two indicating well-being.

The SPSS program v 25 (Armonk, NY, USA) was used to process the data and the *p*- value < 0.05 was considered significant.

## Results

Responding practices were medium sized, mostly with two, three employees, characteristic of the solo practice setting typical in Hungary (Table 1). Big city practices (42.3%) and teaching practices for GP trainees (19.8%) were overrepresented, while responses from mixed and rural practices had much lower numbers (8.5% and 16%, respectively). Work experience was skewed towards elderly doctors, most common categories being 20–25 years and > 35 years.

**Table 1:** Characteristics of Hungarian practices participating in the PRICOV19 study

**How would you characterize the place of this practice? (percentage, N°)**	
Big (inner)city	42.3% (70)
Suburbs	9.4% (16)
(Small) town	23.9% (9,4 39)
Mixed urban–rural	8.5% (14)
Rural	16% (26)
Educational practices (percentage, N°)	
Educational	19.8% (33)
Non-educational	80.2% (132)
Work experience of general practitioners (years, percentage, N°)	
0–4.99	3.0% (5)
5–9.99	9.1% (15)
10–14.99	10.3% (17)
15–19.99	13.9% (23)
20–24.99	20.0% (33)
25–29.99	12.7% (21)
30–34.99	11.5% (19)
> 35 years	19.4% (32)
Number of patients registered with a practice (mean ± SD)	1855 ± 619 individuals
Number of staff per practice (mean ± SD)	2.68 ± 1.3 individuals

Most practices (83%) experienced limitations related to the building of the practice or the infrastructure (Table 2). 138 respondents (83.7%) in the research noticed a change in their work environment. Among the respondents, only 8% stated the lack of any changes in their workplace.

**Table 2:** Perception of Hungarian general practitioners on the changes COVID-19 caused in their practices (percentage, N°)

Since the COVID-19 pandemic, did you experience any limitations related to the building or the infrastructure of this practice to provide high-quality and safe care?	
None	8.6% (14)
Hardly	7.7% (13)
To a limited extend	39.7% (65)
To a large extend	44% (73)
In this practice, do you currently have walk-in hours when patients can come in for a consultation without making an appointment?	
Yes	67% (110)
No	33% (55)
To what extent this practice uses video consultations before the COVID-19 pandemic?	
Never	84.7% (140)
Less than once a week	11.7% (19)
Weekly	3.6% (6)
Daily	0% (0)
Multiple times a day	0% (0)
To what extent this practice uses video consultations since the COVID-19 pandemic?	
Never	63.3% (104)
Less than once a week	15.8% (26)
Weekly	11.2% (19)
Daily	7.1% (12)
Multiple times a day	2.6% (4)
Is a protocol been used in this practice when answering phone calls from potential COVID-19 patients?	
No	15.5% (25)
Yes, this protocol is based on a government guideline	70.1% (116)
Yes, this protocol is not based on a government guideline	14.4% (24)
When answering these phone calls, how often is this protocol used in this practice?	
Never	0% (0)
Rarely	1.2% (2)
Sometimes	1.8% (3)
Regularly	52.8% (87)
Always	44.2% (73)
In every consultation room of a GP in this practice, the most recent information on how to refer a patient to a triage station is immediately available (e.g. procedure, telephone numbers…)	
No, the GP can look for this information on a public website	36.7% (61)
Yes, this is available in print	18.3% (30)
Yes, this is electronically available	43.9% (72)
Other	1.1% (2)
Since the COVID-19 pandemic, staff members are more involved in the triage of patients (by phone, when entering the practice, …)	
Strongly disagree	2.1% (3)
Disagree	4.2% (6)
Neutral	4.7% (7)
Agree	47.9% (79)
Strongly agree	41.1% (70)
Since the COVID-19 pandemic, staff members are more involved in giving information or explaining what a caregiver has said to illiterate patients, patients with low health literacy or migrants	
Strongly disagree	3.5% (6)
Disagree	4.6% (8)
Neutral	11% (18)
Agree	48.6% (80)
Strongly agree	32.4% (53)
Since the COVID-19 pandemic, staff members are more involved in actively reaching out to patients that might postpone healthcare	
Strongly disagree	5.9% (10)
Disagree	18.9% (31)
Neutral	23.8% (39)
Agree	35.1% (58)
Strongly agree	16.2% (27)
Since the COVID-19 pandemic, my responsibilities in this practice increased	
Strongly disagree	0% (0)
Disagree	1.2% (2)
Neutral	1.8% (3)
Agree	22.5% (37)
Strongly agree	74.2% (123)
I do not feel prepared for the task shifting in my professional role since the COVID-19 pandemic	
Strongly disagree	15.9% (27)
Disagree	43.2% (70)
Neutral	18.8% (31)
Agree	18.2% (30)
Strongly agree	4% (7)
I need further training for these amended responsibilities since the COVID-19 pandemic	
Strongly disagree	15.6% (26)
Disagree	25.6% (42)
Neutral	21.1% (35)
Agree	31.7% (52)
Strongly agree	6.1% (10)
I am happy with the task shifting in my professional role since the COVID-19 pandemic	
Strongly disagree	16.9% (28)
Disagree	38.3% (63)
Neutral	20.8% (34)
Agree	19.7% (33)
Strongly agree	4.4% (7)

Despite the epidemic one-third of the practices maintained the possibility of consultation without an appointment which was a significant shift from the routine before the epidemic, when GP consultation without an appointment was widely common in Hungary.

Before the COVID-19 epidemic, video consultation was rare in primary care. The pandemic brought a significant breakthrough in the application of telemedicine techniques, including video communication. Still, 63.3% of practices did not use this form of telemedicine. On the other hand, when communicating through telephone call, 97% of the questioned practices (160) used the specified protocols during telephone consultations. The use of takeaway leaflets became somewhat common (22.4%), especially when the GP had more work experience (Spearman correlation, *r* = 0.21, *p* = 0.007).

The frequency of updating the practice website was positively correlated with information transferring to another colleague of the files that need follow-up (*p* = 0.034; *r* = 0.253) and the existence of video consultations (*p* = 0.019; *r* = 0.258), and negatively with the perception of government assistance (*p* = 0.008; *r* = −0.292).

The role of non-physician employees significantly increased in the triage of patients (89% agree or strongly agree), keeping contact with patients with low health literacy (81% agree or strongly agree). Although to a lesser extent, staff members were more involved in actively reaching out to patients that might postpone healthcare and keeping contact with illiterate patients (51.3% agreeing).

Experienced physicians adjusted to the new challenges: they managed to organize work distribution and adapted to new tasks more easily. Work experience was positively associated with work distribution in case of absence (*p* < 0.001; *r* = 0.295), being happy with the task shifting in their professional role (*p* = 0.007; *r* = 0.218). Moreover, experienced doctors saw central measures more positively: work experience correlated with the perception of having adequate governmental support (*p* = 0.003; *r* = 0.226), and there was a negative correlation between work experience and the feeling that guidelines imposed by the government pose a threat to the well-being of staff, and good organization of practices (*p* < 0.001; *r* = −0.262; *p* = 0.003; *r* = −0.234, respectively).

Most physicians (96.7%, (160)) reported an increase in tasks during the epidemic. According to their own assessment, the new tasks hit PC workers unexpectedly and partially unprepared. At the same time, the new tasks had an alarming effect on PC workers. PC employees reported that additional training would have been necessary to meet these new challenges (37.8% agreeing).

Multiple factors influenced satisfaction with the task shifting in the professional role. It was positively correlated with the distribution of tasks without compromising colleagues’ well-being (*p* < 0.001, *r* = 0.395), neighborhood practice cooperation (*p* = 0.012; *r* = 0.193), perception of government assistance (*p* < 0.001; *r* = 0.288), and work experience (*p* = 0.004; *r* = 0.218), while negatively correlated with questions regarding the guidelines posing a threat to practice organization (*p* = 0.009; *r* = −0.204) and the well-being of employees (*p* < 0.001; *r* = −0.260), and feeling not prepared for the task shifting (*p* = 0.014; r = −0.185).

The mean Mayo Clinic Well-being score was 3.0 ± 2.67 points, 69% experiencing at least some level of distress.

We performed multivariate logistic regression analysis to test which factors are independently associated with the wellbeing of GPs. Dependent variable was having low or elevated distress based on the MCWI scale. The cutoff point of ≥ 2 was used, Low scores indicating well-being were coded as 1, two or higher MCWI scores as 2. Predictor variables for the multivariate analysis were chosen based on the possible scale of impact on practice workflow. Central measurement had a strong influence both on patients' life and on the workflow of practices (Variables: 1, Guidelines imposed by the government are a threat to the organization of this practice, 2, … to the personal well-being of the staff, 3, Adequate support is provided by the government). Information transfer was a key element of care during lockdown both in traditional form (4, this practice has a leaflet with information on COVID-19) and modern form (5, the website of this practice is updated frequently). During the waves of pandemic workload increased significantly (6, Month of questionnaire completion). In univariate analysis work experienced emerged as a central factor (7, Work experience). As practices were more often teaching ones located in cities these factors were included in the analysis for adjustment (8, Location of practice, 9, Presence of trainee).

In multivariate logistic regression analysis (Table 3), *the guidelines imposed by the government on PC practices as a consequence of COVID-19 pose a threat to the personal well-being of the staff* was associated with lower well-being, while *having a takeaway leaflet with information on COVID-19,* and *longer work experience* were positively associated with better well-being. The model was statistically significant (χ ^2^(5) = 29.019, *p* < 0.001), explained 56.1% (Nagelkerke R^2^) of the variance in having a better MCWI and correctly classified 81.0% of cases.

**Table 3:** Multivariate logistic regression analysis assessing well-being of general practitioner during COVID-19

Variable	Odds ratio	95%CI for odds ratio	p
The guidelines imposed by the government pose a threat to the personal well-being of the staff	3.494	1.476−8.269	**0.004**
Does this practice have a leaflet with information on COVID-19 to give to patients?	0.229	0.056−0.941	**0.041**
In the 12 months before the COVID-19 pandemic how often was the information on the website of this practice updated?	1.67	0.903−3.089	0.102
Month of questionnaire completion	0.42	0.151−1.17	0.097
Work experience in general practice (groups of 5 years)	0.558	0.317−0.981	**0.043**

Backward stepwise logistic regression, variables in the final step equation. Dependent variable was high vs low well-being (Mayo Well-being index ≥ 2). Independent variable entered on step 1 were: a) The guidelines imposed by the government pose a threat to the good organization of this practice. b) The guidelines imposed by the government on PC practices pose a threat to the personal well-being of the staff. c) Adequate support is provided by the government. d) Does this practice have a leaflet with information on COVID-19 to give to patients? e) In the 12 months before the COVID-19 pandemic, how often was the information on the website of this practice updated? f) Month of questionnaire completion. g) Work experience in general practice (groups of 5 years). h) How would you characterize the place of this practice? i) This practice has a GP trainee

## Discussion

The pandemic tested the integrity of medical management worldwide [11, 26, 37]. The appropriate response to the epidemic had to be given at different healthcare system levels. The authorities worldwide were forced to adapt to the new challenges, which appeared in part at the level of public health, epidemiology, PC, and inpatient care. In this article, we focused on the changes that occurred in Hungarian PC and examined its effects on the quality of life of GPs in this sector. This report is based on the Hungarian results of the PRICOV-19 research, conducted in 38 countries [26, 38, 39]. The present results were obtained under the supervision of the Hungarian members of the research consortium founded by Ghent University, namely the Department of Family Medicine at Semmelweis University.

The 165 complete questionnaires were filled out by GPs working in solo practices still typical in Hungary. As a consequence of chain-sampling starting out from the Department of Family Medicine respondents' practices were average sized and from all sorts of settlements, but they were most commonly located in big cities (70 respondents), often with an educational background (19.8%, N°:33). Higher willingness to participate from big cities was also present in other national studies in PRICOV-19 [39].

Participating physicians indicated substantial changes in their practices as a consequence of the pandemic. The pre-pandemic generally accepted approach of encounters without scheduling declined, and one third of practices did not accept patients without making an appointment. Safe patient flow was often obstructed by infrastructure or building constraints at least to a limited extent in 83.7% of practices. Two practices working in the same room in shifts is still common in cities; rooms other than the main office are often not available. Lockdown measures, infection control and infrastructure constraints all forced patient care towards telemedicine. The number of personal contacts decreased drastically [25, 32, 33, 40]; home visits were omitted if possible and replaced by new electronic contact forms [4, 41–43].

The increase in the video consultation rate was also substantial in Hungary [44]. This form of keeping contact rarely existed before the pandemic. In our study, 87.3% of respondents claimed not to have had video consultations previously to COVID-19, which dropped to 63.3%. From none of the practices using video consultation, the daily use increased to 9.7% during the pandemic. Similar changes were registered in a number of other participating countries. In Israel video consultation rate increased from 1.3% to 10.5%; in Norway from 2.4% to 33.6%. While this change was important, it also have to be mentioned that the real winner of communication during the pandemic in Hungary was conversation via phone and/or email [45, 46]. These consultation types did not require special devices and were already known by the elderly population [8], being more vulnerable to COVID-19 infection [41, 46, 47]. Telephone consultation could be challenging also to doctors due to the lack of physical examination and personal impressions. Protocols for phone consultations can help minimize the risk of misunderstanding. Most practices used such protocol (84.5%), dominantly the one based on government guidelines. The cloud-based medical services introduced in 2020 further fostered these forms of contact, making it easier for patients and their relatives to access the prescribed medicines [42, 48] and for doctors to immediately obtain the results of consultations and tests [49].

In the office, infection control became a central element of care. Triaging patients by practice staff, detecting cases of covid was part of routine care as indicated by 89% of respondents. This process was often helped by readily available information in 63.3% of practices.

There was a moderate positive correlation between frequent updates of websites, use of video consultation solutions, and the provision of more precise information transfer. There seemed to be two groups of doctors: one group was satisfied with received support, relied mostly on traditional ways of information transfer and refrained from video consultations, while the other group tried to reduce personal consultations and increase video consultations and preferred precise and modern information transfer.

New work structures tended to sometimes multiply existing tasks, not simplifying the work of those working in PC. Online consultations, email requests were an extra task, as PHC system members could not answer them during the consulting hours. Since there was no dedicated specialist for this task at small practices, colleagues were able to resolve this new insistent task only after their duty. These tasks were not only demanding but often put a huge responsibility on the physician making decisions. The vast majority of respondents (96.7%) indicated an increase in their responsibilities. While this change was welcomed by a quarter of physicians (24.1%), many expressed not being prepared for this shift (22.2%), or the need for further training (37.8%).

It is important to note that the most intensive phase of vaccination organization coincided with the questionnaire period, with PC participants also involved in the effort. Due to limited vaccine availability, elderly populations were prioritized, and authorities issued complex eligibility criteria while patients often expressed preferences for specific vaccines. Although EU-procured vaccines were scarce, Sinopharm and Gamaleya Institute vaccines were available. Organizing vaccinations and negotiating with patients required substantial effort in addition to routine care, often resulting in significant overtime for staff in direct patient contact. In contrast, older employees engaged primarily in telemedicine found the task more manageable, highlighting the value of telemedicine and task sharing.

Based on our results, mainly colleagues with greater professional experience had higher resilience during the epidemic. They managed work distribution easier and saw new professional challenges positively. Their view on central measures and the role of the government was less negative.

Meeting the new challenges was difficult for PC and other healthcare workers, who often reported depression of symptoms of anxiety [25, 26, 29, 33], which could deteriorate quality of life. In multivariate analysis, beside information provision, a less negative view of central measures and work experience emerged as positive determinants of better Mayo Clinic Well-being Index determinants. The date of filling out the questionnaire, the location of the practice, or the presence of a GP trainee did not influence well-being. The changes imposed by covid were less likely to be divergent in different settlement types, however, the lack of effect of a trainee is surprising: the help of a young colleague during troubled times could have been of great help. However, during the waves of pandemic resident family doctors were directed to active covid wards, and thus often they were not present in their practices. Greater work experience was accompanied by feelings of competence and less negative views. These physicians could be a core member of providing care during challenging times.

Work experience proved to have a protective effect in our study, resonating with the results described by other PRICOV-19 papers [26]. Their results demonstrated that the older colleagues more easily accepted the fluctuation of unplanned changes, which could have been more challenging for providers with modest work experience.

The questionnaires were completed during April—August 2021, a period with varying workloads in practices. The third wave of the epidemic occurred in March—April 2021, which was one of the most intensive regarding morbidity and mortality indicators [19, 20, 38]. The vaccination campaign also started at the same time, which required the active participation of PC in the organization and carry out of the vaccination campaign. Accordingly, PC practices were exposed to a double load in March and demanded constant overtime from the sector employees. In the summer period, the intensity of duties decreased due to the decrease in the intensity of the epidemic and the organizational tasks related to the vaccination campaign. However when analyzed monthly, the score on the well-being index did not show any significant differences during these periods. The quality of life seems to be a complex phenomenon which is not determined by only the amount of workload. The explanation can be traced back to several reasons: probably the overloaded and therefore most vulnerable colleagues did not deal with the questionnaire at all, or only later in the summer period when the work pressure had already decreased. On the other hand, burnout due to exhausting work is long-lasting, so we could not measure this in a relatively short cross-sectional study.

Based on our findings, primary care was capable of quick adaptation. Cooperation between practices, readiness for technological changes, and continuous training may facilitate this process, in which experienced physicians should play a leading role. Central measurement should be unambiguous and focus on overcoming infrastructural difficulties.

### Strengths and limitations

The strengths of the present study were the number of GPs reached and the questionnaires used. The response rate and the number of physicians filling out the questionnaire were comparable to the rate observed in other European countries. The PRICOV-19 had been validated during the development phase and was successfully translated into multiple languages and used effectively throughout Europe. The MCWI is a structured and validated tool widely used in studies.

This study has some limitations. While chain-sampling made it possible to reach a relatively large number of practices even during the pandemic, such convenient sampling has some pitfalls. We could not collect data on practices that chose not to participate; thus, it is impossible to judge the presence or degree of non-response bias. Unfortunately, a high level of nonresponse was observed across all participating countries in the study [39]. It cannot be excluded that GPs participating in the study were less stressed or burned out or had lower workloads. Those not responding might have experienced more difficulties, and we might have measured slightly more favorable well-being indices. Most aspects of care (patient flow, infection control), however, were based on guidelines, thus huge differences are unlikely between practices.

Self-reported data is prone for social bias; respondents might have given answers that pictures them in a more favorable way. This drawback is present in all studies assessing experiences, opinions, and difficulties.

Contacting GPs on the mailing list of the Department of Family Practice as a first step in the chain-sampling resulted in slightly skewed results. Teaching practices were overrepresented. In Hungary, about 9% of practices are teaching practices, while among respondents this proportion was 19.8%. City practices were also overrepresented (42.3%) compared to the proportion of rural practices (16%). Lower burnout has been reported in teaching practices [50], which might influence our findings. On the other hand, it is unlikely that rural vs city practices had very different settings during the COVID pandemic in terms of policies, opportunities, or duties. Most changes (the rise of telemedicine, vaccination campaigns) were independent of practice locations. Some differences were present in the full dataset of PRICOV in terms of information provision, infrastructural changes, or prescription patterns. However, in Norway, there was no difference between rural and urban practices [51], while in Poland, [52] they only differed in caring for deprived patients.

The chain-sampling made it possible to gather responses during the pandemic, and participants could share their insights immediately, thus we consider recall bias as unlikely to having had a great impact on our results.

Due to the cross-sectional design, we cannot draw conclusions on the cause-and-effect relationship between practice workflow and well-being of physicians, or on the long-term importance of different factors.

## Conclusions

The COVID-19 pandemic brought a substantial change in primary car in Hungary, in terms of patient flow, information exchange and communication. While the feeling of being not prepared and lower well-being was common among GPs, adaptation in task shifting and protocol driven telemedicine appeared, especially among more experienced physicians. The process of adjustment in challenging times could be fostered by unambiguous central measurements and through adequate training.

## Data Availability

All data are centrally stored on the server at Ghent University (Belgium). Researchers from partnering institutions are able to access the non-identifiable dataset. A reasonable request is required to access non-identifiable data by users who are external to the PRICOV-19 consortium. Access will be subject to a data transfer agreement and following approval from the principal investigator of the PRICOV-19 study.

## Abbreviations

GP: General practitioner
PC: Primary care
MCWI: Mayo Clinic Wellbeing Index
PHC: Primary health care
PRICOV-19: Quality and safety in PRImary care in times of COVid-19
